# First Case of *CD40LG* Deficiency in Ecuador, Diagnosed after Whole Exome Sequencing in a Patient with Severe Cutaneous Histoplasmosis

**DOI:** 10.3389/fped.2017.00017

**Published:** 2017-02-10

**Authors:** Luis Alberto Pedroza, Nina Guerrero, Asbjørg Stray-Pedersen, Cristina Tafur, Roque Macias, Greta Muñoz, Zeynep Coban Akdemir, Shalini N. Jhangiani, Levi B. Watkin, Ivan K. Chinn, James R. Lupski, Jordan S. Orange

**Affiliations:** ^1^Colegio de ciencias de la salud-Hospital de los Valles, Universidad San Francisco de Quito, Quito, Ecuador; ^2^Instituto de Microbiología, Universidad San Francisco de Quito, Quito, Ecuador; ^3^Norwegian National Unit for Newborn Screening, Oslo University Hospital, Oslo, Norway; ^4^Department of Pediatrics, Oslo University Hospital, Oslo, Norway; ^5^Hospital Pediátrico Baca Ortiz, Quito, Ecuador; ^6^Baylor-Hopkins Center for Mendelian Genomics, Department of Molecular and Human Genetics, Baylor College of Medicine, Houston, TX, USA; ^7^Department of Molecular and Human Genetics, Baylor College of Medicine, Houston, TX, USA; ^8^Center for Human Immunobiology, Texas Children’s Hospital, Department of Pediatrics, Baylor College of Medicine, Houston, TX, USA; ^9^Department of Pediatrics, Section of Immunology, Allergy, and Rheumatology, Baylor College of Medicine, Texas Children’s Hospital, Houston, TX, USA; ^10^Human Genome Sequencing Center, Baylor College of Medicine, Houston, TX, USA; ^11^Department of Pediatrics, Baylor College of Medicine, Texas Children’s Hospital, Houston TX, USA

**Keywords:** CD40LG, hyper-IgM syndrome, histoplasmosis, whole exome sequencing, primary immunodeficiency diseases

## Abstract

Severe infections with *Histoplasma capsulatum* are commonly observed in patient with secondary immunodeficiency disorders. We report a two and a half years old boy previously healthy with disseminated cutaneous histoplasmosis. Using whole exome sequencing, we found an *indel* mutation at the *CD40LG* gene, suggesting a diagnosis of hyper-IgM (HIGM) syndrome, even in the absence of the usual features for the disease. Interestingly, the patient lives in a region endemic for histoplasmosis. The unusual infections in our case suggest that in children with severe histoplasmosis and resident in endemic areas, HIGM syndrome should be considered as a diagnosis.

## Introduction

**Figure 1:**
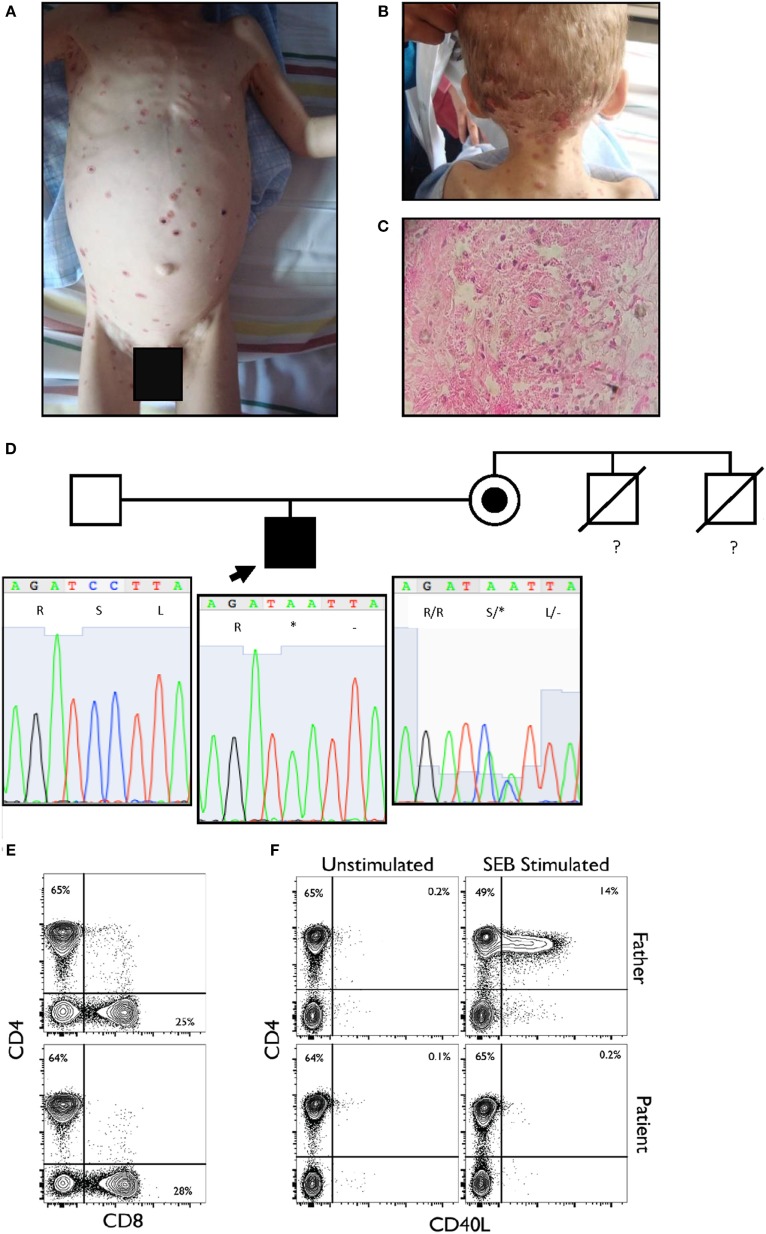
**(A,B)** Disseminated vesicular or dry erythematous and ulcerous lesions over the body and scalp of the proband. **(C)** Histological analysis of the dermis showed intense edema, necrosis of the dermal fibers, and pseudo-granulomatous tissue. A few giant cells and a polymorphonuclear infiltrate are observed. Macrophages are present, loaded with oval-shaped parasites, between 2 and 4 µm in size, positive for periodic acid–Schiff staining (intense red-violet), consistent with *Histoplasma capsulatum*. **(D)** Pedigree of the family sequenced in this study. The arrow indicates the proband. The maternal uncles died of unspecified infections before they reached two years of age. The lower panel shows the Sanger sequencing results and familial segregation for the mutation in CD40LG in the affected proband and both unaffected parents. The mutation is inherited from the mother. **(E)** Normal CD4 and CD8 percentages and ratio within the CD3^+^ population. **(F)** Complete absence of CD40L upregulation in patient CD3^+^ population when stimulated for 6 h with SEB as compared to healthy paternal sample.

We report a two and a half years old boy from a rural tropical region of Ecuador, who presented with a 6-month history of neck and scalp skin lesions. These papules gradually evolved to become pustular and then xerotic, erythematous-ulcerations (Figures 1A,B). Lesional biopsy was consistent with cutaneous histoplasmosis (Figure 1C). We initially ruled out secondary immunodeficiencies, including HIV, raising the suspicion of a primary immunodeficiency disease (PIDD).

During the course of his evaluation, our patient was also identified as having mucous-containing diarrhea positive for *Cryptosporidium parvum*, a pulmonary infection with tracheal aspirates yielding *Pseudomonas aeruginosa* and *Candida albicans*, as well as oral lesions consistent with candidiasis. He additionally had hepatomegaly 3 cm below the costal margin and diffuse lymphadenopathy.

Laboratory studies demonstrated normal white blood cell counts and lymphocyte subpopulations (CD3^+^: 2,410 cells/μL, CD3^+^CD4^+^: 1,501 cells/μL, CD3^+^CD8^+^: 845 cells/μL, and CD19^+^: 682 cells/μL). Serum immunoglobulin levels were as follows: IgG, 251 mg/dL (480–1200 mg/dL); IgA, below detectable limits (33–180 mg/dL); and IgM, 51 mg/dL (54–200 mg/dL).

Amphotericin B, 1 mg/kg/day was provided for 4 weeks, and intravenous immunoglobulin (600 mg/kg) was administered. After a 2-month hospitalization, the patient was discharged with a prophylactic therapy regimen consisting of trimethoprim/sulfamethoxazole (80/400 mg) every 3 days, itraconazole (100 mg) daily for 2 months and then every 3 days for an additional year, and IVIG 400 mg/kg every 6 weeks. For the past 2 years, the patient has been free of infection with an approximate IgG trough level between 409 and 560 mg/dL.

The patient is the only son of unrelated and healthy parents. Family history was notable for two maternal uncles who died in childhood from pneumonia and diarrhea—suggesting an X-linked recessive trait for susceptibility to severe infections.

The patient’s infections, altered laboratory values and both personal and familial history, suggested a PIDD, probably an X-linked combined immunodeficiency. Given the lack of tools to further investigate the immunology of our patient at our center in Ecuador, as well as the relatively broad differential diagnosis surrounding combined immunodeficiency and *Histoplasma* susceptibility, we performed whole exome sequencing (WES) as a family trio (patient, mother, and father) as part of an ongoing collaboration with the Baylor-Hopkins Center for Mendelian Genomics (1). Exome capture was performed with the in-house developed BCM-HGSC Core design (52 Mb; Roche NimbleGen, Madison, WI, USA), as previously described (2). The variant calling was performed by the ATLAS2 suite (3), identifying a consecutive dinucleotide substitution at the exon 2 of the *CD40LG* in the proband (Table 1), as the most probable variant associated with our patient’s phenotype. This mutation creates a premature stop codon (NM_000074:exon2:c.233_234delinsAA:p.S78*), predicted to result in the loss of the entire extracellular portion of the protein (including the TNF-like domain required for the interaction with CD40) or a complete loss-of-function allele due to mRNA instability and nonsense-mediated decay as predicted by our algorithm. This variant was not reported at ExAC database at the time of the publication and was confirmed by Sanger sequencing in the family trio (Figure 1D). The mother was a healthy carrier, and the variant thus segregated with the family history. Other gene variant information from the WES data of the affected individual is listed in Table 1. While this variant would represent a novel mutation, it suggests the diagnosis of hyper-IgM (HIGM) syndrome, and it is consistent with several reported nonsense or frameshift mutations in early codons associated with *CD40LG* deficiency (4). Induced expression of CD40L after staphylococcal enterotoxin B superantigen stimulation was examined, and complete lack of expression of the protein by the CD4^+^ T cells of the patient was observed (Figures 1E,F).

**Table 1:** **Variant information**. The dinucleotide substitution is predicted by conceptual translation to result in a PTC at position 78. The bioinformatics prediction suggests a likely disease causing mutation associated with HIGM1.

Gene name	*CD40LG*	*CD40LG*
Chromosome	chrX	chrX
Position (hg19)	135732501	135732502
Sequence: reference/alternative	C/A	C/A
Proband: reference/alternative	4/160	4/168
Mother: reference/alternative	163/126	162/125
Father: reference/alternative	147/0	143/0
Mutation type	Indel	
Refseq	NM_000074	
Mutation: cDNA	c.233_234delinsAA	
Mutation: protein	p.Ser78*	
Predicted effect	Premature truncating codon (PTC), disease causing	
Associated phenotype/MIM number	Immunodeficiency with hyper-IgM, type 1; HIGM1/308230	

## Background and Discussion

Hyper-IgM is a well-known PIDD caused by defects in class-switch recombination and/or B cell costimulation. It commonly presents with *Pneumocystis jirovecii* pneumonia and *Cryptosporidium parvum* diarrhea or more standard consequences of hypogammaglobulinemia (4). Our patient presented with diarrhea and pneumonia, and we confirmed the presence of *Cryptosporidium parvum* in the feces. Susceptibility to the microorganism isolated from the tracheal aspirate, *P. aeruginosa* and *Candida albicans*, while not common in HIGM, has been previously reported (5, 6). Interestingly, our patient presented with a low serum IgM level, and although the disease name suggests high levels of IgM, in at least 50% of cases of *CD40LG* deficiency, the IgM level can be normal or even low (7).

The main presentation in our patient was the histoplasmosis, although uncommon in HIGM, it has been previously reported. Interestingly, these cases have been reported in areas considered endemic for histoplasmosis (8): the American cases near to the Mississippi River Valley (9, 10) and the Latin-American in Brazil and Argentina (5, 11). Additional factors such as bird contact, presence of guano soil, or farming activities inside the endemic areas seem to be important (8). Remarkably, our patient family lives in an isolated rural region in the north of Ecuador with exclusive dedication to farming. Since *Histoplasma* infections even inside endemic areas are commonly observed in patients with risk factors like HIV or old males after years of smoking (8); in children without risk factors and severe histoplasmosis, who live in endemic areas, HIGM may represent a more likely presentation for this diagnosis. Also while lymphadenopathy in HIGM is rare, our patient was likely to have this as a manifestation of the histoplasmosis. Unfortunately, it was not possible to biopsy the lymph nodes or the liver and thus we were not able to confirm this. Nevertheless, it has been previously reported that in endemic areas, children with histoplasmosis could develop the infection in the peripheral lymph nodes (12), suggesting again that environmental influences are essential, and thus the need to think about PIDD from a geographical region-specific perspective.

## Concluding Remarks

We present detailed clinical findings in a patient with X-linked HIGM due to a novel *indel* mutation in *CD40LG*, for whom the diagnosis was confirmed after WES was performed. The unusual infections in our case suggest that in children with severe histoplasmosis, resident in endemic areas, HIGM should be considered as a diagnosis.

## Ethics Statement

This study was performed with parental permission and approval by the Institutional Review Board for Baylor College of Medicine and Affiliated Hospitals and The Institutional Review Board of the USFQ (Universidad San Francisco de Quito).

## Author Contributions

Conception and design of the work: LP, AS-P, JL, and JO. Clinical data collection: LP, NG, CT, RM, and GM. Genomic data collection and analysis: IC, ZA, SJ, and JL. Laboratory data collection and analysis: LP, IC, and LW. Drafting the article: LP, NG, CT, RM, GM, AS-P, LW, and IC. Critical revision of the article: LP, AS-P, LW, IC, ZA, JL, and JO. Final approval of the version to be published; agreement to be accountable for all aspects of the work in ensuring that questions related to the accuracy or integrity of any part of the work are appropriately investigated and resolved: LP, NG, AS-P, CT, RM, GM, ZA, SJ, LW, IC, JL, and JO.

## Conflict of Interest Statement

The authors declare that the research was conducted in the absence of any commercial or financial relationships that could be construed as a potential conflict of interest.
